# Relationship between disproportionately enlarged subarachnoid-space hydrocephalus and white matter tract integrity in normal pressure hydrocephalus

**DOI:** 10.1038/s41598-023-48940-6

**Published:** 2023-12-04

**Authors:** Sunju Lee, Jae-Sung Lim, E-nae Cheong, Yoojin Lee, Jae Woo Kim, Ye Eun Kim, Sungyang Jo, Hyung-Ji Kim, Woo Hyun Shim, Jae-Hong Lee

**Affiliations:** 1 Department of Neurology, Seosan Jungang General Hospital, Seosan-si, Chungcheongnam-do Republic of Korea; 2 Department of Neurology, Asan Medical Center, University of Ulsan College of Medicine, 88 Olympic-ro 43-gil, Sonpa-gu, Seoul, 05505 Republic of Korea; 3 Department of Medical Science and Asan Medical Institute of Convergence Science and Technology, Asan Medical Center, University of Ulsan College of Medicine, Seoul, Republic of Korea; 4 Department of Neurology, Uijeongbu Eulji Medical Center, Eulji University School of Medicine, Uijeongbu, Republic of Korea; 5 Department of Radiology, Asan Medical Center, University of Ulsan College of Medicine, Seoul, Republic of Korea

**Keywords:** Dementia, Hydrocephalus, Neurodegenerative diseases, Neurological disorders

## Abstract

Normal pressure hydrocephalus (NPH) patients had altered white matter tract integrities on diffusion tensor imaging (DTI). Previous studies suggested disproportionately enlarged subarachnoid space hydrocephalus (DESH) as a prognostic sign of NPH. We examined DTI indices in NPH subgroups by DESH severity and clinical symptoms. This retrospective case–control study included 33 NPH patients and 33 age-, sex-, and education-matched controls. The NPH grading scales (0–12) were used to rate neurological symptoms. Patients with NPH were categorized into two subgroups, high-DESH and low-DESH groups, by the average value of the DESH scale. DTI indices, including fractional anisotropy, were compared across 14 regions of interest (ROIs). The high-DESH group had increased axial diffusivity in the lateral side of corona radiata (1.43 ± 0.25 vs. 1.72 ± 0.25, *p* = 0.04), and showed decreased fractional anisotropy and increased mean, and radial diffusivity in the anterior and lateral sides of corona radiata and the periventricular white matter surrounding the anterior horn of lateral ventricle. In patients with a high NPH grading scale, fractional anisotropy in the white matter surrounding the anterior horn of the lateral ventricle was significantly reduced (0.36 ± 0.08 vs. 0.26 ± 0.06, *p* = 0.03). These data show that DESH may be a biomarker for DTI-detected microstructural alterations and clinical symptom severity.

## Introduction

Idiopathic normal pressure hydrocephalus (iNPH) is a neurodegenerative disease characterized by the classical Hakim’s triad of gait disturbance, cognitive impairment, and urinary disturbance in patients with ventriculomegaly in the absence of elevated cerebrospinal fluid (CSF) pressure^1^ Disproportionately enlarged subarachnoid-space hydrocephalus (DESH) has been reported to help predict neurologic improvement after shunt surgery^2–5^. However, the clinical utility of DESH remains controversial, as some meta-analysis reports showed poor diagnostic performance of DESH regarding predicting the treatment response in iNPH^6,7^. Thus, it is still unclear whether DESH truly represents changes in the clinical presentation and structural connectivity of these patients.

Diffusion tensor imaging (DTI) has been widely applied to various clinical conditions to evaluate microstructural changes in brain white matter^8^. Because it allows early detection of microstructural changes in white matter fibers noninvasively, DTI also has been widely applied to iNPH^9^. Pure compressive lesion without stretch was associated with decreased fractional anisotropy (FA), axial diffusivity (AD), increased mean diffusivity (MD), and radial diffusivity (RD)^10^. Meanwhile, stretch and distortion without compression were related to increased AD and decreased FA and RD^9^. However, previous studies did not explore the differences in DTI indices depending on the severity of DESH. Only one study explored the association between DTI indices and DESH findings; however, only FA was compared in the internal capsule and the corpus callosum between patients with DESH and without DESH^11^. DESH was associated with cerebral white matter compression and blockage of physiologic CSF circulation^11^. Cerebral blood flow studies have revealed decreased perfusion in white matter adjacent to ventricles compared to subcortical white matter in patients with iNPH^12,13^. Moreover, periventricular brain tissue is distinguished by neuronal degeneration and gliosis, presumably due to altered extracellular fluid dynamics^14^. Therefore, studying the correlation of DESH with structural changes in the brain using DTI will be helpful in understanding where and how important structural changes occur that determine clinical symptoms or treatment responses.

The aim of our study was to evaluate the associations between DTI indices and clinical scores, including DESH and iNPH grading scales (iNPHGS) in iNPH patients. We hypothesized that (1) patients with higher DESH scores would have more unfavorable findings in DTI indices than those with lower DESH scores, and (2) the triad of iNPH symptoms would be associated with microstructural damage to major whiter matter tracts.

## Results

### Study population

From January 2018 to December 2020, 39 inpatients met the diagnostic criteria for NPH and underwent DTI. Of these, three patients were excluded due to a history of traumatic brain injury and ICH, and an additional three patients were excluded due to technical errors during DTI preprocessing. As a result, 33 patients were finally included in the analysis. For the control group, there were 149 patients with a CDR of 0 who underwent DTI during the period, including the 33 patients matched by age, sex, and education to the NPH group.

### Demographics, clinical and cognitive performance

Patients with iNPH were more often accompanied by diabetes mellitus (iNPH 60.6% vs. control 21.2%, *p* < 0.01); however, there were no significant differences between the two groups in age, sex, education years, and hypertension. The iNPH group exhibited lower scores on the MMSE, GDS, CDR, and CDR-SB than the control group (Table 1). Compared to the control group, the iNPH group exhibited a statistically significant increase in Evan’s index, third ventricle width, and DESH scale while demonstrating a decrease in callosal angle (Table 1). Mean DTI values in iNPH patients and controls for fourteen ROIs were presented in the supplementary file (Supplementary Table S4). In summary, the iNPH group had significantly lower FA and higher MD, AD, or RD values compared with the control group, including bilateral centrum semiovale, corona radiata, posterior limb of the internal capsule, PVWM adjacent to anterior and posterior horns of lateral ventricle, genu of the corpus callosum, and splenium of the corpus callosum.

**Table 1:** Demographics and baseline characteristics of the patients.

	Low DESH (n = 14)	High DESH (n = 19)	*p* value	Control (n = 33)	iNPH (n = 33)	*p* value*
Age, years	75.8 ± 4.9	74.5 ± 4.9	0.451	74.8 ± 5.2	75.0 ± 4.8	Matched
Male	8 (57.1%)	11 (57.9%)	1.000	19 (57.6%)	19 (57.6%)	Matched
Education, years	8.5 ± 5.9	10.3 ± 5.1	0.334	9.7 ± 5.3	9.6 ± 5.4	Matched
HTN	10 (71.4%)	12 (63.2%)	0.901	16 (48.5%)	22 (66.7%)	0.181
Diabetes mellitus	7 (50.0%)	13 (68.4%)	0.478	7 (21.2%)	20 (60.6%)	0.006
Cognitive tests scores						
MMSE	24.5 [19.0; 25.0]	24.0 [18.5; 25.0]	0.674	28.0 [26.0; 29.0]	24.0 [19.0; 25.0]	< 0.001
GDS	4.0 [3.0; 5.0]	4.0 [3.0; 5.0]	0.627	2.0 [2.0; 2,0]	4.0 [3.0; 5.0]	< 0.001
CDR	0.8 [0.5; 1.0]	1.0 [0.5; 1.0]	0.414	0.0 [0.0; 0.0]	1.0 [0.5; 1.0]	< 0.001
CDR sum of boxes	3.3 [1.6; 5.4]	4.8 [3.4; 6.5]	0.108	0.0 [0.0; 0.0]	4.5 [2.0; 6.0]	< 0.001
DESH scale	6.0 [4.3; 6.0]	8.0 [7.0; 9.0]	< 0.001	1.0 [0.0; 2.0]	7.0 [6.0; 8.0]	< 0.001
VM score	1.0 [1.0; 2.0]	2.0 [2.0; 2.0]	0.034	0.0 [0.0; 0.0]	2.0 [1.0; 2.0]	< 0.001
Dilated SF score	1.0 [1.0; 2.0]	2.0 [2.0; 2.0]	0.081	0.0 [0.0; 1.0]	2.0 [1.0; 2.0]	< 0.001
Tight HC score	1.0 [0.3; 1.0]	2.0 [1.0; 2.0]	0.005	0.0 [0.0; 1.0]	1.0 [1.0; 2.0]	< 0.001
Acute CA score	0.0 [0.0; 1.9]	2.0 [1.0; 2.0]	< 0.001	0.0 [0.0; 1.0]	1.0 [0.0; 2.0]	< 0.001
Focal SD score	1.0 [1.0; 1.0]	1.0 [1.0; 1.0]	0.442	1.0 [0.0; 1.0]	1.0 [1.0; 1.0]	< 0.001
Evans index	0.35 ± 0.05	0.38 ± 0.04	0.052	0.27 ± 0.03	0.37 ± 0.04	< 0.001
Callosal angle, degree	106.1 ± 11.6	83.0 ± 12.8	< 0.001	117.3 ± 12.7	92.8 ± 16.5	< 0.001
3rd ventricle width, mm	13.2 ± 3.5	14.5 ± 2.4	0.246	8.6 ± 1.9	13.9 ± 3.0	< 0.001
CSF tap test, n	10	16				
TUG time before tap test, sec	15.4 $\pm$ 6.5	27.2 $\pm$ 16.5	0.019			
CSF tap test responders^†^						
6-h post-tap TUG	5/10 (50.0%)	9/16 (56.3%)	0.999			
24-h post-tap TUG (cumulative)	7/10 (70.0%)	10/16 (62.5%)	0.999			
MMSE $\ge$ 3 points	2/10 (20.0%)	3/16 (18.8%)	0.999			
Ventriculoperitoneal shunt, n	1	7				
Post-operative changes in iNPHGS						
− 10 points	0/1 (0%)	1/7 (14.3%)				
− 3 points	0/1 (0%)	1/7 (14.3%)				
− 2 points	1/1 (100.0%)	4/7 (57.1%)				
− 1 point	0/1 (0%)	1/7 (14.3%)				
Complications, n	0/1 (0%)	2^‡^/7 (28.6%) valve malfunction				

Data represent the mean ± standard deviation or median and interquartile ranges. Group differences were assessed using Student's t-test or Mann–Whitney U test for continuous variables and χ^2^ test for dichotomous variables.

*iNPH* idiopathic normal pressure hydrocephalus, *HTN* hypertension, *DM* diabetes, *K-MMSE* Korean version of mini-mental status examination, *GDS* global deterioration scale, *CDR* clinical dementia rating, *DESH* disproportionately enlarged subarachnoid space hydrocephalus, *VM* ventriculomegaly, *SF* sylvian fissure, *HC* high convexity, *CA* callosal angle, *SD* sulcal dilation, *TUG* timed up and go.

*Comparisons between NPH and its paired controls were evaluated with paired t-tests or Mann–Whitney U tests for matched pairs and McNemar's test.

^†^Defined as an improvement of 10% or more in time spent in a TUG evaluation.

^‡^These two patients improved after shunt revision.

**Figure 1:**
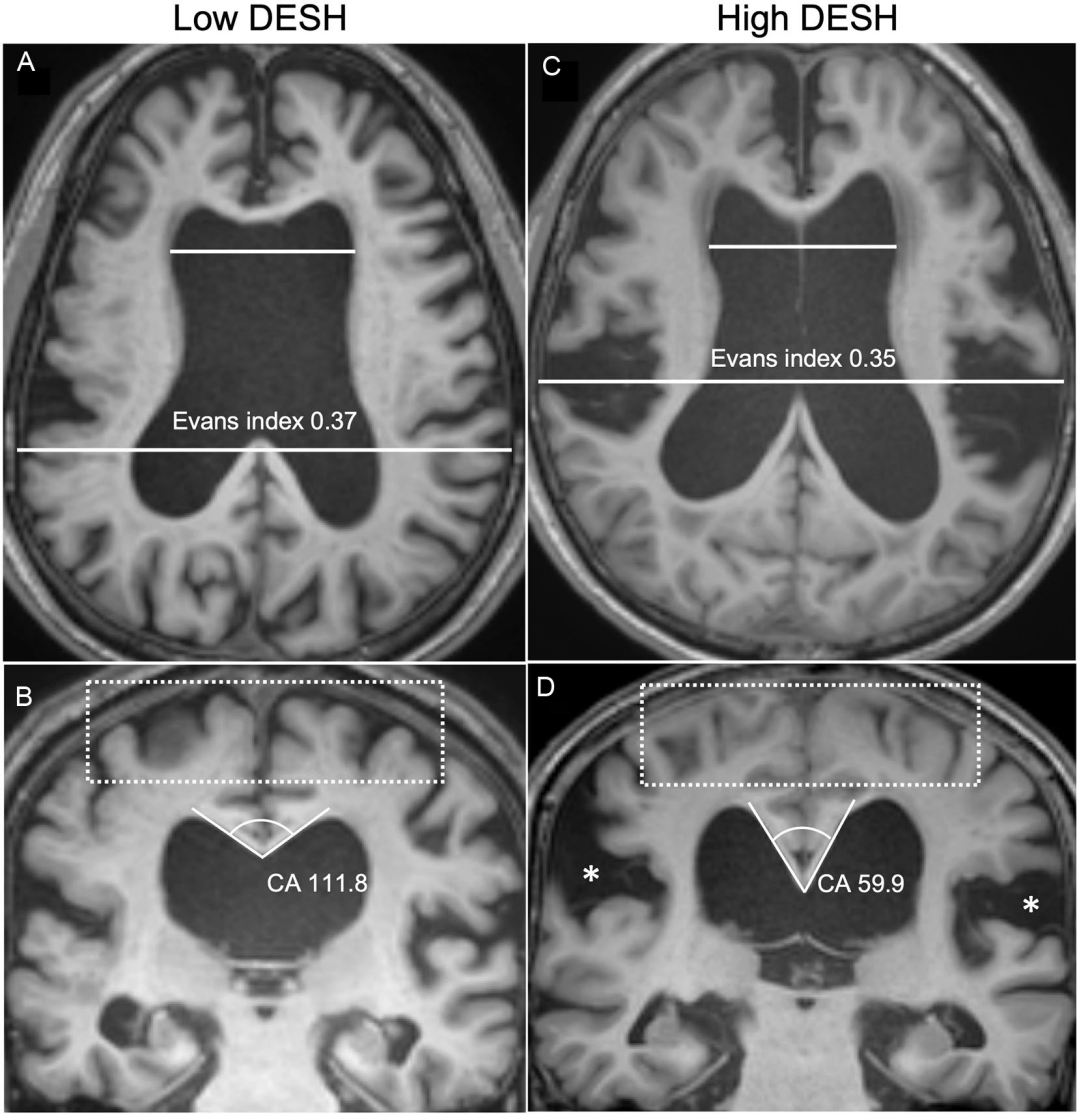
Representative MR images of the high-DESH and low-DESH group. MR coronal and axial images of representative patients in the low-DESH (**a**,**b**) and high-DESH (**c**,**d**) groups. In the low-DESH patient, the Evans index is increased to 0.37 (grade 2, panel **a**), the sylvian fissures are slightly enlarged (grade 1), but no tight high convexity is observed, (white-dotted rectangle) and the callosal angle is 111.8°, which is above 90° (panel **b**). This patient has a DESH score of 3. In the high-DESH patient, the Evans index is increased to 0.35 (grade 2, panel **c**), similar to the low-DESH patient, but in addition, the sylvian fissure is markedly dilated bilaterally (grade 2, white asterisks), tight convexity is seen (grade 2, white-dotted rectangle), and the callosal angle is less than 90° at 59.9° (grade 2). This patient also has focal sulcal dilatation (grade 1, not shown), resulting in a DESH score of 9. *DESH* disproportionately enlarged subarachnoid space hydrocephalus, *CA* callosal angle.

Patients with iNPH were divided into two subgroups, high DESH (DESH scale ≥ 7, n = 19) vs low DESH (DESH scale ≤ 6, n = 14), by the average value (6.73) of the DESH scale (Fig. 1). There was no significant difference in demographics, risk factors, cognitive scores, and iNPHGS between the high- and low-DESH groups (Table 1). The CSF tap test was performed in 10 and 16 patients in the low- and high-DESH groups, respectively. Based on the criterion of at least 10% improvement in gait velocity in the timed up and go evaluation^15^, 70% (low-DESH group) and 62.5% (high-DESH group) patients showed improvement, respectively. Improvements of 3 or more points in cognitive function assessed by Mini-Mental State Examination (MMSE) before and after the CSF tap test were observed in approximately 20 and 18.8%, respectively^16^. Shunt surgery was performed in only 8 of 33 patients, all but one of whom were in the high-DESH group. Two patients required reoperation due to shunt malfunction, but all patients had improved postoperative subjective symptoms and iNPHGS (Table 1). Due to the retrospective nature of the study, objective quantitative measures were not consistently collected before and after surgery. DTI was also not collected postoperatively.

### DTI values in DESH subgroups

The high-DESH subgroup had a significantly lower callosal angle than the low-DESH subgroup, and the Evans index tended to be higher. Compared to the low DESH subgroup, the high DESH subgroup demonstrated significant differences in ventriculomegaly, tight high convexity, and acute callosal angle.

DTI values in DESH subgroups for fourteen ROIs were presented in Table 2. In high-DESH groups, FA values were decreased, or MD, AD, or RD was increased compared with those in low-DESH groups in the anterior and lateral side of corona radiata and PVWM adjacent to the anterior horns of the lateral ventricle. After multiple comparison corrections, only AD in the lateral side of corona radiata showed a significant difference between the two groups.

**Table 2:** Diffusion tensor imaging analysis in disproportionately enlarged subarachnoid space hydrocephalus groups.

ROIs	FA				MD				AD				RD			
	Low DESH (n = 14)	High DESH (n = 19)	*p*	q*	Low DESH (n = 14)	High DESH (n = 19)	*p*	q*	Low DESH (n = 14)	High DESH (n = 19)	*p*	q*	Low DESH (n = 14)	High DESH (n = 19)	*p*	q*
Centrum semiovale																
Lt	0.382 ± 0.058	0.357 ± 0.066	0.259	0.759	0.817 [0.749;0.947]	0.946 [0.800;1.070]	0.271	0.422	1.147 [1.050;1.402]	1.316 [1.066;1.441]	0.397	0.762	0.658 [0.603;0.784]	0.761 [0.648;0.871]	0.255	0.502
Rt	0.378 ± 0.083	0.336 ± 0.060	0.100	0.467	0.878 [0.783;0.967]	0.965 [0.846;1.138]	0.132	0.285	1.296 ± 0.213	1.345 ± 0.187	0.490	0.762	0.705 [0.570;0.794]	0.839 [0.682;0.947]	0.065	0.303
Corona radiata, anterior side																
Lt	0.328 ± 0.071	0.308 ± 0.090	0.498	0.759	0.928 [0.854;1.092]	1.061 [0.920;1.279]	0.091	0.285	**1.337 ± 0.158**	**1.470 ± 0.201**	**0.049**	0.343	0.754 [0.678;0.947]	0.889 [0.743;1.120]	0.186	0.434
Rt	0.311 ± 0.081	0.290 ± 0.082	0.452	0.759	0.940 [0.849;1.005]	1.089 [0.941;1.218]	0.163	0.285	1.345 ± 0.238	1.421 ± 0.183	0.312	0.762	0.800 [0.690;0.841]	0.906 [0.753;1.066]	0.174	0.434
Corona radiata, lateral side																
Lt	**0.327 ± 0.064**	**0.279 ± 0.053**	**0.026**	0.217	**1.124 ± 0.208**	**1.268 ± 0.161**	**0.033**	0.231	1.521 ± 0.224	1.652 ± 0.197	0.084	0.392	**0.926 ± 0.206**	**1.075 ± 0.152**	**0.023**	0.287
Rt	0.264 ± 0.058	0.269 ± 0.068	0.830	0.830	**1.126 ± 0.231**	**1.333 ± 0.221**	**0.014**	0.196	**1.433 ± 0.249**	**1.719 ± 0.246**	**0.003**	**0.042**	**0.972 ± 0.227**	**1.140 ± 0.221**	**0.041**	0.287
Post limb of internal capsule																
Lt	0.7482 [0.702;0.771]	0.7474 [0.718;0.770]	0.529	0.749	0.808 ± 0.062	0.774 ± 0.068	0.148	0.285	1.630 ± 0.101	1.618 ± 0.119	0.777	0.777	0.360 [0.320;0.435]	0.334 [0.321;0.388]	0.287	0.502
Rt	0.757 [0.737;0.780]	0.748 [0.723;0.770]	0.653	0.762	0.782 [0.755;0.806]	0.766 [0.753;0.779]	0.114	0.285	1.623 [1.613;1.691]	1.617 [1.558;1.637]	0.226	0.762	0.335 [0.324;0.386]	0.338 [0.321;0.381]	0.815	0.878
Ant horn of lateral ventricle																
Lt	0.343 ± 0.086	0.330 ± 0.043	0.651	0.762	0.942 [0.832;1.262]	0.968 [0.886;1.061]	0.928	0.928	1.327 [1.191;1.680]	1.330 [1.205;1.432]	0.760	0.777	0.780 [0.670;1.054]	0.809 [0.744;0.875]	0.900	0.900
Rt	**0.360 ± 0.067**	**0.300 ± 0.081**	**0.031**	0.217	0.957 [0.924;1.069]	1.005 [0.964;1.194]	0.132	0.285	1.362 [1.285;1.464]	1.389 [1.261;1.529]	0.733	0.777	0.780 [0.711;0.833]	0.874 [0.767;1.028]	0.091	0.318
Post horn of lateral ventricle																
Lt	0.415 ± 0.090	0.424 ± 0.079	0.768	0.827	0.952 [0.826;1.124]	1.078 [0.893;1.186]	0.506	0.596	1.444 [1.287;1.558]	1.598 [1.330;1.751]	0.439	0.762	0.724 [0.599;0.872]	0.777 [0.719;0.916]	0.506	0.617
Rt	0.463 ± 0.071	0.446 ± 0.082	0.529	0.759	0.975 ± 0.184	1.023 ± 0.2007	0.495	0.596	1.487 ± 0.252)	1.524 ± 0.228	0.667	0.777	0.719 ± 0.167	0.773 ± 0.209	0.433	0.606
Corpus callosum																
Genu	0.741 [0.626;0.779]	0.706 [0.43;0.755]	0.377	0.759	0.922 [0.849;1.131]	0.964 [0.881;1.064]	0.553	0.596	1.914 [1.842;0.252]	1.891 [1.815;2.009]	0.553	0.774	0.455 [0.364;0.714]	0.494 [0.399;0.628]	0.529	0.617
Splenium	0.724 ± 0.136	0.750 ± 0.107	0.542	0.759	0.847 [0.765;0.996]	0.810 [0.777;0.907]	0.553	0.596	1.847 ± 0.235	0.781 ± 0.178	0.362	0.762	0.363 [0.277;0.581]	0.375 [0.254;0.477]	0.397	0.606

Data represent the mean ± standard deviation or median and interquartile ranges. Student t-test or Mann–Whitney U test were performed to compare DTI parameters in ROIs between two groups.

Significant values are in [bold].

*DTI* diffusion tensor imaging, *DESH* disproportionately enlarged subarachnoid space hydrocephalus, *ROI* region of interest, *iNPH* idiopathic normal pressure hydrocephalus, *FA* fractional anisotropy, *MD* mean diffusivity, *AD* axial diffusivity, *RD* radial diffusivity, *Lt* left, *Rt* right.

*Adjusted for multiple comparison corrections using the False Discovery Rate (FDR) method.

### DTI values in iNPHGS subgroups

Patients with iNPH were also categorized into two subgroups by the average value (5.39) of total iNPHGS: low-iNPHGS (iNPHGS ≤ 5, n = 20) and high-iNPHGS (iNPHGS ≥ 6, n = 13) subgroups. There were significant differences in sex, MMSE, GDS, CDR, and CDR sum of boxes between the high and low iNPHGS groups (Supplementary Table S5). DTI values in iNPHGS subgroups for fourteen ROIs were presented in Table 3. In the high-iNPHGS group, FA, MD, or AD values were decreased compared with those in the low-iNPHGS group in the PVWM adjacent to the anterior horns of the lateral ventricle and posterior limb of the internal capsule. After multiple comparison corrections, FA in the anterior horns of the lateral ventricle showed a significant difference between the two groups.

**Table 3:** Diffusion tensor imaging (DTI) analysis in idiopathic normal pressure hydrocephalus grading scale (iNPHGS) subgroups.

ROIs	FA				MD				AD				RD			
	Low iNPHGS (n = 20)	High iNPHGS (n = 13)	*p*	q*	Low iNPHGS (n = 20)	High iNPHGS (n = 13)	*p*	q*	Low iNPHGS (n = 20)	High iNPHGS (n = 13)	*p*	q*	Low iNPHGS (n = 20)	High iNPHGS (n = 13)	*p*	q*
Centrum semiovale																
Lt	0.366 ± 0.062	0.371 ± 0.067	0.830	0.900	0.827 [0.767;1.031]	0.946 [0.792;1.074]	0.573	0.761	1.159 [1.047;1.412]	1.316 [1.066;1.469]	0.413	0.578	0.685 [0.614;0.824]	0.761 [0.595;0.869]	0.899	0.899
Rt	0.366 ± 0.081	0.335 ± 0.057	0.244	0.854	0.936 ± 0.180	0.999 ± 0.180	0.337	0.744	1.307 ± 0.205	0.350 ± 0.187	0.552	0.703	0.751 ± 0.181	0.832 ± 0.181	0.270	0.756
Corona radiata, anterior side																
Lt	0.311 ± 0.086	0.326 ± 0.078	0.606	0.863	1.001 [0.856;1.279]	1.038 [0.936;1.100]	0.703	0.786	0.415 ± 0.229	1.411 ± 0.129	0.955	0.955	0.804 [0.678;1.120]	0.866 [0.760;0.947]	0.899	0.899
Rt	0.300 ± 0.087	0.296 ± 0.073	0.888	0.900	0.941 [0.873;1.242]	1.089 [0.957;1.142]	0.372	0.744	1.380 ± 0.242	1.402 ± 0.148	0.766	0.825	0.785 [0.698;1.079]	0.884 [0.810;1.009]	0.434	0.798
Corona radiata, lateral side																
Lt	0.314 ± 0.066	0.278 ± 0.049	0.105	0.490	1.154 ± 0.201	1.288 ± 0.154	0.050	0.243	1.544 ± 0.224	1.677 ± 0.182	0.084	0.329	**0.959 ± 0.199**	**1.093 ± 0.147**	**0.045**	0.630
Rt	0.274 ± 0.068	0.256 ± 0.057	0.439	0.863	1.204 ± 0.286	1.309 ± 0.152	0.184	0.515	1.556 [1.247;1.826]	1.639 [1.521;1.780]	0.181	0.422	1.031 ± 0.276	1.127 ± 0.146	0.206	0.721
Post limb of internal capsule																
Lt	0.760 [0.708;0.771]	0.733 [0.714;0.771]	0.676	0.863	0.784 [0.765;0.841]	0.746 [0.724;0.797]	0.052	0.243	**1.654 ± 0.101**	**1.575 ± 0.110**	**0.042**	0.294	0.349 [0.317;0.405]	0.334 [0.330;0.393]	0.650	0.899
Rt	0.761 [0.723;0.775]	0.748 [0.729;0.758]	0.548	0.863	**0.779 [0.765;0.804]**	**0.755 [0.731;0.778]**	**0.007**	0.098	**1.634 [1.614;1.673]**	**1.569 [1.528;1.620]**	**0.005**	0.070	0.339 [0.325;0.385]	0.337 [0.324;0.367]	0.456	0.798
Ant horn of lateral ventricle																
Lt	0.355 ± 0.083	0.305 ± 0.066	0.079	0.490	0.964 [0.880; 1.106]	0.923 [0.852;1.058]	0.730	0.786	1.343 [1.229;1.520]	1.208 [1.153;1.408]	0.094	0.329	0.800 [0.641;0.913]	0.809 [0.699;0.883]	0.813	0.899
Rt	**0.358 ± 0.075**	**0.257 ± 0.060**	**0.002**	**0.028**	0.976 [0.941;1.109]	0.991 [0.962;1.142]	0.524	0.761	1.403 [1.310;1.485]	1.298 [1.239;1.484]	0.372	0.578	0.781 [0.689;0.934]	0.874 [0.802;0.971]	0.158	0.721
Post horn of lateral ventricle																
Lt	0.425 ± 0.092	0.413 ± 0.068	0.678	0.863	1.023 [0.814;1.136]	1.055 [0.909;1.184])	0.598	0.761	1.451 [1.293;1.668]	1.520 [1.287;1.734]	0.624	0.728	0.742 [0.599;0.925]	0.777 [0.723;0.866]	0.413	0.798
Rt	0.463 ± 0.076	0.439 ± 0.079	0.389	0.863	0.959 ± 0.190	1.069 ± 0.194	0.116	0.406	1.462 ± 0.246	1.579 ± 0.207	0.168	0.422	0.636 [0.553;0.852]	0.799 [0.717;0.851]	0.147	0.721
Corpus callosum																
Genu	0.720 [0.673;0.768]	0.671 [0.571;0.758]	0.524	0.863	0.957 [0.873;1.088]	0.941 [0.866;1.084]	0.957	0.957	1.922 [1.848;2.049]	1.859 [1.798;1.978]	0.221	0.442	0.479 [0.392;0.636]	0.529 [0.389;0.629]	0.730	0.899
Splenium	0.737 ± 0.141	0.742 ± 0.077	0.900	0.900	0.795 [0.762;0.983]	0.847 [0.783;0.919]	0.598	0.761	1.834 ± 0.235	1.770 ± 0.142)	0.383	0.578	0.325 [0.254;0.570]	0.394 [0.278;0.456]	0.650	0.899

Data represent the mean ± standard deviation or median and interquartile ranges. Student t-test or Mann–Whitney U test were performed to compare DTI parameters in ROIs between two groups.

Significant values are in [bold].

*DTI* diffusion tensor imaging, *iNPHGS* idiopathic normal pressure hydrocephalus grading score, *ROIs* regions-of-interest, *FA* fractional anisotropy, *MD* mean diffusivity, *AD* axial diffusivity, *RD* radial diffusivity, *Lt* left, *Rt* right.

*Adjusted for multiple comparison corrections using the False Discovery Rate (FDR) method.

In addition, patients with iNPH were subclassified into two subgroups by the average scores (1.75) of each clinical domain: low GD (n = 18) versus high GD (n = 15), low CI (n = 14) versus high CI (n = 19), low UD (n = 15) versus high UD (n = 18). Clinical variables and DTI values of subgroups for each GD, CI, and UD were presented in the supplementary material (Supplementary Tables S5–S8).

## Discussion

This study investigated the differences in DTI indices according to DESH score and clinical symptom severity in patients with iNPH. Our data showed that bilateral corona radiata and PVWM adjacent to the right anterior horn of the lateral ventricle were associated with the severity of DESH. Clinical severity was related to the posterior limb of the internal capsule and PVWM adjacent to the anterior horn of the lateral ventricle. However, after multiple comparison corrections, only AD in the lateral side of the corona radiata showed a significant difference between the low- and high-DESH groups and FA in the anterior horns of the lateral ventricle showed a significant difference between the low- and high-iNPHGS groups.

Our results provide important clues to how structural changes of DESH are associated with clinical symptoms. A previous study investigated the differences in DTI indices in patients with DESH compared to those without^11^ studies have yet explored the difference in DTI indices according to the severity of DESH. Reduced FA, increased MD, AD, or RD can be interpreted as a pressure effect on the cerebral white matter; therefore, the pressure on the white matter appears to be greater in NPH patients with high DESH than in those with low DESH. The findings are consistent with previous studies comparing patients with iNPH to controls^17^. These results suggest that DTI metrics may represent a pathologic process that worsens with increasing disease severity. Although the pathophysiology of DESH is not fully understood, FA can be decreased as the white matter is compressed, and MD can be increased due to transependymal diffusion and edema by pressure effect^10,11^. More specifically, decreases of FA in bilateral corona radiata could be interpreted as vertical compression resulting from ventricular enlargement on horizontal projection of corona radiata and increases in MD and RD might reflect transependymal diffusion^10,18,19^. In addition, the vertical compression of PVWM by ventricular dilation might reduce blood flow or stasis of CSF and interstitial fluid. Then, this process disrupts periventricular projection fibers to the cerebral cortex, which are closely related to neurological symptoms in patients with NPH. Based on these findings, our results suggest that the pressure effects following the severity of DESH depend on their proximity to the ventricle. For this reason, the DTI indices in the PVWM adjacent to the anterior horn of the lateral ventricle or corona radiata close to the ventricular walls may have shown significant differences compared to other ROIs^20^. The reason behind the predominance of observed changes in the anterior-located ROIs in the frontal lobes is yet to be determined.

All our patients were symptomatic iNPH, and the differences in DTI indices according to the severity of the symptoms were also compared. As a result, values of FA, MD, or AD were decreased in the high-iNPHGS group compared with the low-iNPHGS group in the PVWM adjacent to the anterior horns of the lateral ventricle and posterior limb of the internal capsule. The directional of changes in MD and AD was opposite to the results comparing the DESH subgroups, with MD and AD decreasing as the symptom severity increased. In previous studies, AD was reported to reflect axonal degeneration, and its reduction was associated with axonal injury^21–23^. Thus, it can be interpreted that low AD values in the high-iNPHGS group compared to those in the low-iNPHGS group might reflect more severe axonal injury that leads to prominent clinical presentation. The MD values were defined as the average molecular diffusion rates within the voxel and were calculated by the formula (AD + 2RD)/3 so that the decreased AD value can lead to decreased MD values^24^. Regarding the location of the damage, our analysis showed that significant differences in DTI indices were mainly observed in the bilateral posterior limb of the internal capsule and the PVWM adjacent to the bilateral anterior horn of the lateral ventricle. The corticospinal tract passes through the posterior limb of the internal capsule, and the anterior thalamic radiation projects through the PVWM adjacent to the bilateral anterior horn of the lateral ventricle respectively. Our results suggest that the dysfunction of these major pathways may be associated with the expression of clinical symptoms in iNPH patients. To support this hypothesis, additional research is required to ascertain how changes in DTI metrics correspond to the amelioration of symptoms following surgery.

We further evaluated DTI indices in subgroups of each symptom of Hakim’s triad. There were significant differences in DTI indices between subgroups in the bilateral posterior limbs of the internal capsule, the left corona radiata, the PVWM adjacent to the bilateral anterior horns of the lateral ventricles, and the right posterior horn of the lateral ventricles, which are associated with the corticospinal tract, anterior thalamic radiation, and inferior longitudinal fasciculus, respectively. In a previous study, gait disturbances in patients with iNPH were associated with alterations in FA values in the corticospinal tract and anterior thalamic radiations projecting to the supplementary motor areas. Cognitive impairment evaluated by MMSE, CDR, and frontal assessment battery, including trail-making test-A, was associated with changes in FA, AD, and RD in the frontal and parietal subcortical white matter, including anterior thalamic radiation^9,25–28^. The urinary disturbance was related to the loss of voluntary control of bladder contractions due to stretched periventricular sacral fibers of the corticospinal tracts^29^. Taken together, ventricular expansion may compress the neuronal connectivity or vascular structures of the periventricular tissues, and this compression may disrupt white matter tracts such as anterior thalamic radiation, inferior longitudinal fasciculus, and corticospinal tract, which are involved in cognition, gait, and micturition^29–31^. The relationship between decreased AD and MD values, which were abnormally elevated in the posterior limb of the internal capsule and inferior longitudinal fasciculus, and symptomatic improvement after shunt surgery suggests that DTI metrics may be useful surrogate markers to reflect the pathophysiology of iNPH, such as cerebral white matter compression^10,11^.

It is also worth noting that, as previously discussed, there were no significant differences in response to the CSF tap test based on the DESH subgroup^6,7^. After careful screening, 7 out of 8 patients who underwent ventriculoperitoneal shunt operation were high-DESH patients, and two of these patients required shunt revision but had symptomatic improvement after surgery. However, postoperative DTI changes that could support the hypothesis of this study were unfortunately not collected due to the retrospective nature of the study. Changes in DTI metrics that may support postoperative symptomatic improvement are a topic to be explored in future well-designed large-scale studies.

This study has several limitations. First, all ROIs were manually derived; such ROI-based analyses can be affected by the subjectivity of the investigator, and ROI derivation is time-consuming. In previous studies, various DTI analysis techniques have been applied, including manual ROI placement, tract-specific analysis (TSA), and tract-based spatial statistics (TBSS), depending on the characteristics of the patient population^9,19,26,28,32–37^. Other alternative standardization methods, such as TBSS or peak width of skeletonized mean diffusivity, were also considered in our study but were challenging to apply to the iNPH patients in this study due to their substantial structural variations^38,39^. In this investigation, a neurologist with over five years of experience consistently drew ROIs by referring to more detailed structural images, such as T1 and T2, and predefined anatomical landmarks. This issue has been discussed in detail as a limitation in previous studies^20,32,40,41^. Second, the results of multiple comparison corrections were insignificant except for the corona radiata lateral side in the DESH group comparison and the lateral ventricle anterior horn area in the iNPHGS group comparison. Due to the limited number of patients and the exploratory nature of the study, we interpreted the results based on uncorrected *p* values; however, given the potential for type 1 error, the results of this study should be interpreted for hypothesis-generating purposes and replicated in subsequent studies.

Our data showed that bilateral corona radiata and the right anterior horn of the lateral ventricle were significantly associated with the severity of DESH. Clinical severity was related to periventricular white matter adjacent to the anterior horn of the lateral ventricle and posterior limb of the internal capsule. These results might suggest the disruption of the corticospinal tract and anterior thalamic radiation serving the supplementary motor area. DTI is noninvasive and a quick sequence that can be easily added to a routine MRI without contrast agent injection. In addition to visual assessment such as DESH, DTI might allow an early evaluation of microstructural changes and would be a valuable biomarker to reflect the severity of clinical symptoms in iNPH patients.

## Methods

The local institutional review board of the Asan Medical Center endorsed the study protocol. The requirement for informed consent was waived by the ethical committee of the Asan Medical Center due to its retrospective nature and low risk to participants. All methods were performed in accordance with the relevant guidelines and regulations of the Asan Medical Center Ethics Committee and the Declaration of Helsinki.

### Participants

Patients with possible iNPH, according to the diagnostic criteria by the guidelines for the management of iNPH (third edition), who were hospitalized in Asan Medical Center from January 2018 to December 2020 and underwent brain magnetic resonance imaging (MRI), including DTI, were eligible for this study^42^. Patients with secondary hydrocephalus or underlying pathology associated with gait or cognitive impairment, including stroke, head trauma, major depression, or drug abuse, were excluded. The control group was selected from patients who presented to Asan Medical Center in Seoul with a primary symptom of memory decline and underwent neuropsychological evaluations and brain MRI but did not have objective cognitive impairment, a CDR score of 0, and did not meet the diagnostic criteria for iNPH. This group included individuals with normal aging, subjective memory impairment, and transient global amnesia. Each control was matched to the case for age (± 3 years), level of education (± 3 years), and sex. The local institutional review board (IRB) approved the study protocol.

### Clinical Assessment

We assessed the severity of the symptoms of iNPH using the NPHGS (Supplementary Table S1)^43^. The iNPHGS was composed of the following three subdomains, each scored from 0 to 4 (maximum total score of 12): gait disturbance (GD), cognitive impairment (CI), and urinary disturbance (UD). Information on demographics, comorbidities, neuropsychological tests, and neuroimaging characteristics was obtained by reviewing the electronic medical records and the Picture Archiving and Communication System. Neuropsychological assessments were conducted, including the comprehensive neuropsychological battery (Seoul Neuropsychological Screening Battery, 2nd Edition; SNSB-II), which consists of the five domains of cognitive function: attention, language and related functions, visuospatial function, memory, and frontal/executive function, the Korean version of the Mini-Mental State Examination (MMSE), global deterioration scale (GDS), CDR and CDR sum of boxes^44^. We collected whether a CSF tap test was performed and whether symptoms improved afterward. A responder was defined as a patient who showed an improvement of 10% or more in walking speed 6 h and 24 h after the CSF tap test, based on the results of the timed up and go evaluation performed at the time of the tap test^15^. For cognitive function, we checked the change in MMSE score before and after the CSF tap test to determine if the score improved by 3 points or more^16^. We investigated whether a ventriculoperitoneal shunt operation was performed and checked the change in symptoms before and after the operation through the electronic medical records.

### Imaging acquisition

All study patients underwent brain MRI imaging using the 3 T MRI scanner (Philips 3 Tesla Achieva; Philips Healthcare, Eindhoven, The Netherlands). DTI sequence was obtained with a single-shot echo-planar imaging sequence with TR/TE = 10,788/70 ms, flip angle = 90°, FOV, 230 × 230; acquisition matrix, 116 × 116; in-plane acquisition resolution, 0.958 × 0.958 mm; slice thickness, 2 mm; reconstruction matrix, 240 × 240. Diffusion-sensitizing gradients were applied along 32 directions with b value = 1000 s/ mm^2^. According to the eligible criteria, T2 fluid-attenuated inversion recovery, susceptibility–weighted imaging, and T1–weighted images were reviewed to exclude the presence of parenchymal lesions, which could be associated with gait disturbance and cognitive impairment, and measured the DESH score.

### DESH score

The DESH score is composed of the following five items, each scored from 0 to 2 (maximum total score 10): ventriculomegaly, dilated sylvian fissures, tight high convexity, acute callosal angle, and focal sulcal dilation^3^ (Supplementary Table S2). Ventriculomegaly was assessed using the Evans' index (EI), the ratio of the maximum width of the frontal horns to the maximum transverse diameter of the inner table in the same section^45^, measured and calculated manually from the axial T1–weighted MRI. Then, it was graded as follows: 0, EI < 0.3; 1, 0.3 ≤ EI < 0.35; or 2, EI ≥ 0.35. Dilated Sylvian fissures and tight high convexity were evaluated on coronal images and graded as follows, respectively: 0, normal; 1, unilateral dilation; or 2, bilateral dilation; and 0, normal; 1, slight compression; 2, definitive compression^46,47^. The callosal angle (CA) measured on a coronal image is the angle between the lateral ventricle and the posterior commissure and is perpendicular to the anterior–posterior commissure plane^48,49^. CA was graded as follows: 0, CA ≥ 100°; 1, 90 ≤ CA < 100°; 2, CA < 90°. Sulcal dilation was graded as follows: 0, no sulci; 1, some sulci present; or 2, many sulci present. These imaging features of DESH were visually assessed by three neurologists (S Lee, S Jo, and H-J Kim) blind to clinical data. The intraclass correlation coefficient (ICC) for inter-rater reliability of DESH scoring was 0.814.

### DTI processing

The processing of the diffusion tensor images was performed with FMRIB software library (FSL) version 6.0.4 (http://www.fmrib.ox.ac.uk/fsl) ^50^. First, each diffusion tensor image was corrected for head motion and residual eddy current distortion to the reference B0 volume using EDDY_CORRECT. Then, we excluded non-brain tissue from the corrected data with the Brain Extraction Tool (BET). Finally, individual diffusion maps (FA, MD, AD, and RD) were created by fitting a tensor model using the FMRIB Diffusion Toolbox (FDT). In the analysis stages, diffusion values were extracted from predefined regions-of-interest (ROIs) described below, on the diffusion maps. The *fslstats* command was used to extract values, which were used for statistical analysis.

### Generation of ROIs

**Figure 2:**
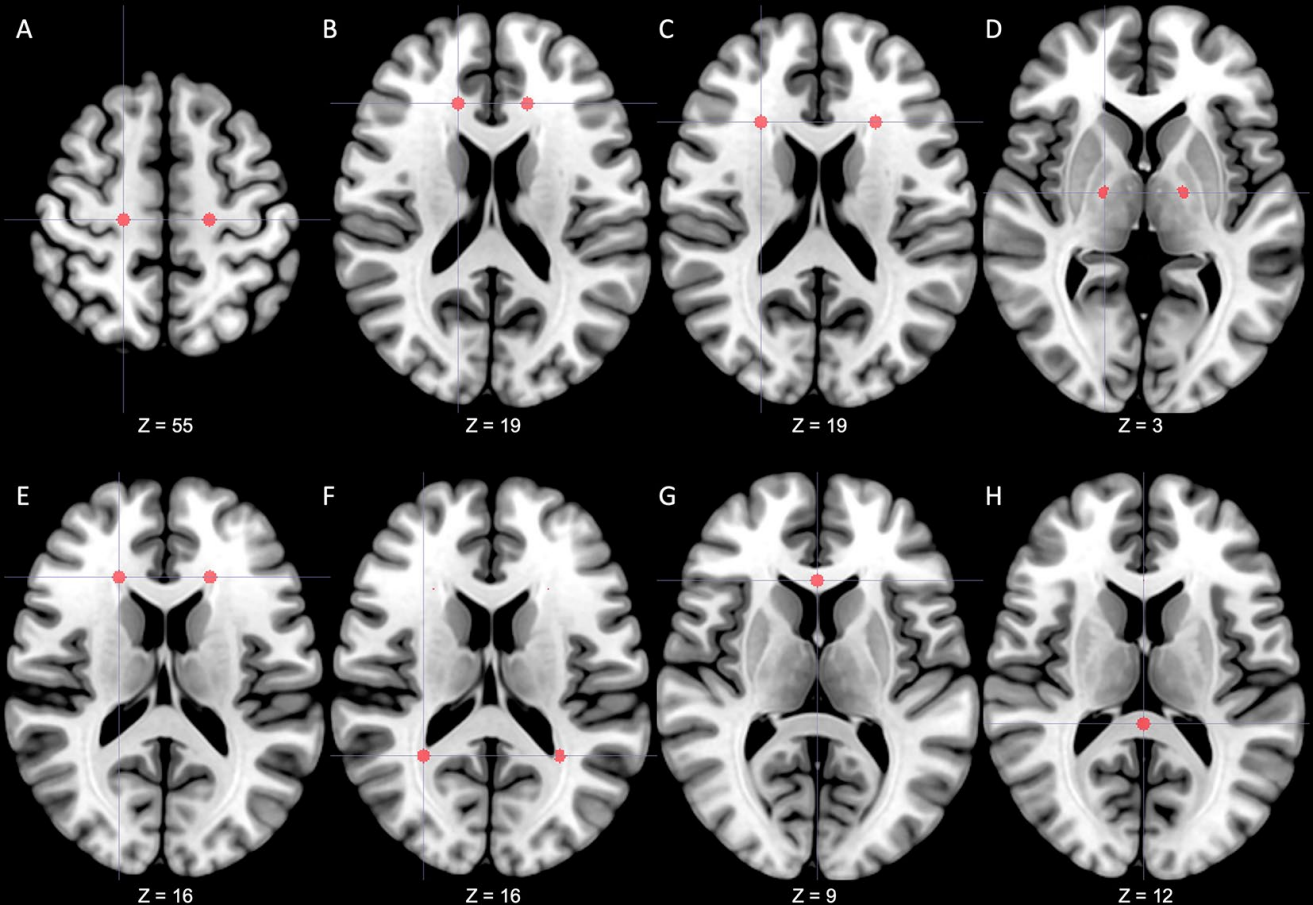
Predetermined regions-of-interest. A total of 14 regions of interest were predetermined in the centrum semiovale (**A**), anterior area of corona radiata (**B**), lateral area of corona radiata (**C**), posterior limbs of the internal capsule (**D**), anterior horns of the lateral ventricle (**E**), posterior horns of the lateral ventricle (**F**), genu of corpus callosum (**G**) and splenium of corpus callosum (**H**). From a radiological perspective, the right side of the figure is the patient's left side. The z coordinate represents the position on the MNI152 standard template. *MNI* Montreal Neurological Institute.

In this study, it was impossible to normalize DTI images into MNI (Montreal Neurological Institute) spaces due to the characteristics of patients with considerable brain deformity. Therefore, fourteen ROIs that showed significant differences in patients with iNPH were predetermined based on the results of previous studies^9,25,26,32,41^. Predetermined regions for ROIs were bilateral centrum semiovale, anterior and lateral areas of corona radiata, posterior limb of the internal capsule, periventricular white matter (PVWM) adjacent to anterior horns (area of anterior thalamic radiation) and posterior horns (area of inferior longitudinal fasciculus) of lateral ventricle, genu and splenium of the corpus callosum. (Fig. 2) The ROI masks were manually drawn using the AFNI (https://afni.nimh.nih.gov/) with a radius of 3 mm in three-dimensional space based on each patient's FA image and adjacent landmarks as references^32,51^. To ensure the objectivity of ROI selection, the MNI152 standard coordinates were first defined (Supplementary Table S3), and each ROI was selected by referring to the corresponding reference coordinates and surrounding anatomical landmarks in each patient's FA image. All ROIs were generated independently of clinical information by an investigator with five years (S Lee) of experience in neurology and double-checked for appropriateness by separate investigators (Y Lee and JW Kim). ROIs were exported to Image J (National Institutes of Health, Bethesda, USA) and applied to all DTI output files^52^. Right and left ROIs were analyzed separately.

### Statistical analysis

Statistical analysis between iNPH cases and matched controls was performed using paired t-test or Mann–Whitney U tests for continuous variables and a McNemar’s test for categorical data. The iNPH group was categorized by the mean value of each DESH and iNPHGS. In addition, we also divided the iNPH group into two subgroups based on the mean score in each domain of the iNPHGS to identify differences in DTI indices by symptom type. Student t-test or Mann–Whitney U tests were performed to compare the two groups' clinical and DTI parameters in predetermined ROIs. The *p* values were adjusted for multiple comparisons using the false discovery rate (FDR) method. Given the limited number of patients and exploratory nature of the study, we presented the uncorrected *p* value as the primary outcome, with the corrected q-value presented for reference. The significance threshold for between-group differences was considered at *p* < 0.05. Analyses were performed with Statistical Package for Social Sciences (SPSS) Version 21.

### Supplementary Information

Supplementary Tables.

## Data Availability

The datasets used and/or analyzed during the current study are available from the corresponding author upon reasonable request.
