# Supplementary material for: Relationship between disproportionately enlarged subarachnoid-space hydrocephalus and white matter tract integrity in normal pressure hydrocephalus
Source: Sci Rep. 2023 Dec 4;13:21328. doi: 10.1038/s41598-023-48940-6 (PMC10694135; doi:10.1038/s41598-023-48940-6)
Supplement: Supplementary file 1 — Supplementary Tables. [file 41598_2023_48940_MOESM1_ESM.docx]

**Table S1. iNPH grading scale**

| Grade | Definition |
| --- | --- |
| Gait disturbance |  |
| 0 | Normal |
| 1 | Complaints of dizziness of drift and dysbasia but no objective gait disturbance |
| 2 | Unstable but independent gait |
| 3 | Walking with any support |
| 4 | Walking no possible |
| Cognitive impairment |  |
| 0 | Within normal range |
| 1 | No apparent dementia but apathetic |
| 2 | Socially dependent but independent at home |
| 3 | Partially dependent at home |
| 4 | Totally dependent |
| Urinary disturbance |  |
| 0 | Absent urinary incontinence |
| 1 | Absent urinary incontinence but with pollakiuria or urinary urgency |
| 2 | Occasional urinary incontinence (1–3 or more times per week but less than once per day) |
| 3 | Continuous urinary incontinence (1 or more times per day) |
| 4 | Bladder function is almost or completely deficient |

The grades of gait disturbance, cognitive impairment, and urinary disturbance are added to obtain the total grade, ranging from 0 to 12.

Abbreviations: iNPH, idiopathic normal pressure hydrocephalus

**Table S2. DESH (disproportionately enlarged subarachnoid space hydrocephalus) scale**

| Grade | Definition |
| --- | --- |
| Ventriculomegaly |  |
| 0 | Evans’ index < 0.3 |
| 1 | 0.3 ≤ Evans’ index < 0.35 |
| 2 | Evans’ index ≥ 0.35 |
| Dilated sylvian fissures |  |
| 0 | Normal or narrow |
| 1 | Slight dilatation or unilateral |
| 2 | Bilateral dilatation |
| Tight high convexity |  |
| 0 | Normal or wider than normal |
| 1 | Slight compression |
| 2 | Definitive compression |
| Acute callosal angle |  |
| 0 | Callosal angle > 100° |
| 1 | 90° < Callosal angle < 100° |
| 2 | Callosal angle < 90° |
| Focal sulcal dilation |  |
| 0 | Not present |
| 1 | Some present |
| 2 | Many present |

**Table S3. MNI coordinates for each region of interest**

| Region of interest | X coordinate | Y coordinate | Z coordinate |
| --- | --- | --- | --- |
| Centrum semiovale | 19 (-19)^*^ | -21 | 55 |
| Corona radiata, anterior | 15 (-15) | 30 | 19 |
| Corona radiata, lateral | 25 (-25) | 22 | 19 |
| Posterior limb of internal capsule | 17 (-17) | -9 | 3 |
| Anterior horn of lateral ventricle | 20 (-20) | 27 | 16 |
| Posterior horn of lateral ventricle | 30 (-30) | -51 | 16 |
| Corpus callosum, genu | 0 | 26 | 9 |
| Corpus callosum, splenium | 0 | -37 | 12 |

^*^ Parentheses indicate coordinates for the left side.

Abbreviations: MNI, Montreal Neurological Institute

**Table S4. Regions-of-interest (ROIs) analysis showing mean DTI values in iNPH group and control group**

|  | | **FA** | | | **MD** | | | **AD** | | | **RD** | | | |
| --- | --- | --- | --- | --- | --- | --- | --- | --- | --- | --- | --- | --- | --- | --- |
| **ROIs** | | **Control**  **(n = 33)** | **iNPH**  **(n = 33)** | **p** | **Control**  **(n = 33)** | **iNPH**  **(n = 33)** | **p** | **Control**  **(n = 33)** | **iNPH**  **(n = 33)** | **p** | **Control**  **(n = 33)** | **iNPH**  **(n = 33)** | **p** |  |
| **centrum semiovale** | **Lt** | **0.513 ± 0.059** | **0.368 ± 0.063** | **<0.001*** | **0.732 [0.702;0.760]** | **0.880 [0.784;1.068]** | **<0.001*** | 1.179 [1.151;1.212] | 1.220 [1.065;1.435] | 0.086 | **0.493 [0.471;0.535]** | **0.700 [0.610;0.864]** | **<0.001*** |  |
|  | **Rt** | **0.536 ± 0.059** | **0.354 ± 0.073** | **<0.001*** | **0.735 [0.718;0.757]** | **0.926 [0.827;1.075]** | **<0.001*** | **1.0201 [1.169;1.278]** | **1.296 [1.165;1.497]** | **0.019*** | **0.490 [0.472;0.521]** | **0.746 [0.640;0.877]** | **<0.001*** |  |
| **corona radiata, anterior side** | **Lt** | 0.335 ± 0.062 | 0.317 ± 0.082 | 0.289 | **0.839 [0.801;0.877]** | **1.031 [0.884;1.148]** | **<0.001*** | **1.134 [1.085;1.217]** | **1.383 [1.296;1.482]** | **<0.001*** | **0.689 [0.651;0.743]** | **0.818 [0.692;0.984]** | **<0.001*** |  |
|  | **Rt** | **0.344 ± 0.064** | **0.299 ± 0.081** | **0.014*** | **0.836 [0.822;0.884]** | **0.967 [0.912;1.206]** | **<0.001*** | **1.142 [1.100;1.212]** | **1.333 [1.251;1.513]** | **<0.001*** | **0.692 [0.649;0.738]** | **0.810 [0.706;1.043]** | **<0.001*** |  |
| **corona radiata, lateral side** | **Lt** | **0.436 ± 0.084** | **0.299 ± 0.062** | **<0.001*** | **0.858 ± 0.084** | **1.207 ± 0.193** | **<0.001*** | **1.293 ± 0.129** | **1.596 ± 0.216** | **<0.001*** | **0.641 ± 0.098** | **1.012 ± 0.190** | **<0.001*** |  |
|  | **Rt** | **0.444 ± 0.070** | **0.267 ± 0.063** | **<0.001*** | **0.858 ± 0.104** | **1.245 ± 0.245** | **<0.001*** | **1.301 ± 0.138** | **1.598 ± 0.282** | **<0.001*** | **0.636 ± 0.106** | **1.069 ± 0.236** | **<0.001*** |  |
| **Posterior limb of internal capsule** | **Lt** | 0.723 [0.709;0.747] | 0.747 [0.714;0.771] | 0.189 | **0.7550 [0.734;0.772]** | **0.778 [0.746;0.837]** | **0.011*** | **1.532 ± 0.072** | **1.623 ± 0.110** | **<0.001*** | 0.367 [0.342;0.387] | 0.346 [0.320;0.394] | 0.986 |  |
|  | **Rt** | **0.733 [0.713;0.753]** | **0.757 [0.724;0.770]** | **0.023*** | **0.740 [0.722;0.768]** | **0.777 [0.755;0.788]** | **0.004*** | **1.510 [1.471;1.555]** | **1.619 [1.561;1.643]** | **<0.001*** | 0.352 [0.330;0.374] | 0.338 [0.324;0.382] | 0.339 |  |
| **anterior horn of lateral ventricle** | **Lt** | **0.454 ± 0.063** | **0.335 ± 0.080** | **<0.001*** | **0.854 [0.812;0.897]** | **0.942 [0.862;1.067]** | **<0.001*** | 1.319 [1.265;1.388] | 1.330 [1.194;1.456] | 0.491 | **0.613 [0.584;0.707]** | **0.804 [0.971;0.890]** | **<0.001*** |  |
|  | **Rt** | **0.462 ± 0.067** | **0.325 ± 0.080** | **<0.001*** | **0.879 [0.820;0.930]** | **0.985 [0.941;1.128]** | **<0.001*** | 1.349 [1.294;1.403] | 1.387 [1.282;1.484] | 0.296 | **0.634 [0.579;0.701]** | **0.824 [0.731;0.966]** | **<0.001*** |  |
| **posterior horn of lateral ventricle** | **Lt** | **0.516 ± 0.063** | **0.420 ± 0.083** | **<0.001*** | **0.871 [0.808;0.924]** | **1.055 [0.881;1.143]** | **0.011*** | 1.441 [1.362;1.550] | 1.520 [1.287;1.669] | 0.537 | **0.576 [0.537;0.660]** | **0.761 [0.678;0.909]** | **<0.001*** |  |
|  | **Rt** | **0.530 [0.484;0.558]** | **0.459 [0.405;0.509]** | **0.003*** | 0.912 [0.852;1.043] | 0.997 [0.839;1.145] | 0.189 | 1.537 ± 0.161 | 1.520 ± 0.235 | 0.504 | **0.639 [0.560;0.713]** | **0.727 [0.584;0.851]** | **0.031*** |  |
| **corpus callosum** | **Genu** | **0.785 [0.733;0.807]** | **0.716 [0.630;0.758]** | **<0.001*** | **0.833 [0.804;0.867]** | **0.950 [0.869;1.084]** | **<0.001*** | **01.797 [1.737;1.922]** | **1.903 [1.822;2.038]** | **0.005*** | **0.339 [0.318;0.422]** | **0.480 [0.389;0.629]** | **<0.001*** |  |
|  | **Splenium** | **0.823 [0.788;0.870]** | **0.762 [0.674;0.837]** | **0.021*** | **0.755 [0.726;0.797]** | **0.822 [0.776;0.921]** | **0.002*** | 1.731 ± 0.150 | 1.809 ± 0.203 | 0.093 | **0.268 [0.209;0.299]** | **0.373 [0.272;0.513]** | **0.012*** |  |

Data represent the mean ± standard deviation or median and interquartile ranges. Comparisons between NPH and its paired controls were evaluated with paired t-tests or Mann-Whitney U tests for matched pairs.

Abbreviations: DTI, diffusion tensor imaging; ROIs, regions-of-interest; iNPH, idiopathic normal pressure hydrocephalus; FA, fractional anisotropy; MD, mean diffusivity; AD, axial diffusivity; RD, radial diffusivity, Lt, left; Rt, right.

**Table S5. Demographics of clinical subgroups**

|  | **low iNPHGS**  **(n = 20)** | **high iNPHGS**  **(n = 13)** | **p** | **low GD score**  **(n = 18)** | **high GD score**  **(n = 15)** | **p** | **low CI score**  **(n = 14)** | **high CI score**  **(n = 19)** | **p** | **low UD score**  **(n = 15)** | **high UD score**  **(n = 18)** | **p** |
| --- | --- | --- | --- | --- | --- | --- | --- | --- | --- | --- | --- | --- |
| Age, years | 74.6 ± 3.9 | 75.8 ± 6.2 | 0.489 | 74.3 ± 3.9 | 75.9 ± 5.8 | 0.336 | 74.2 ± 3.6 | 75.6 ± 5.6 | 0.415 | 74.6 ±3.8 | 75.4 ±5.7 | 0.649 |
| Male | **15 (75. 0%)** | **4 (30.8 %)** | **0.031*** | 11 (61.1 %) | 8 (53.3 %) | 0.923 | 11 (78.6%) | 8 (42.1 %) | 0.082 | 11 (73.3%) | 8 (44.4 %) | 0.187 |
| Education, years | 11.0 ± 4.5 | 7.4 ± 6.2 | 0.064 | 10.5 [6.0;13.0] | 9.0 [6.0;14.0] | 0.798 | 12.0 [6.0;12.0] | 9.0 [5.0-14.5] | 0.555 | **11.9 ± 4.0** | **7.6 ± 5.8** | **0.022*** |
| HTN | 14 (70.0 %) | 8 (61.5 %) | 0.900 | 14 (77.8 %) | 8 (53.3 %) | 0.266 | 10 (71.4 %) | 12 (63.2 %) | 0.901 | 10 (66.7 %) | 12 (66.7 %) | 1.000 |
| Diabetes mellitus | 12 (60. 0%) | 8 (61.5 %) | 1.000 | 11 (61.1 %) | 9 (60.0 %) | 1.000 | 8 (57.1 %) | 12 (63.2 %) | 1.000 | 9 (60.0 %) | 11 (61.1 %) | 1.000 |
| MMSE | **25.0 [22.8; 26.0]** | **19.0 [13.0; 24.0]** | **0.001*** | 24.5 [21.0; 25.0] | 21.0 [16.5; 24.5] | 0.223 | **25.5 [25.0; 26.8]** | **20.0 [15.0; 24.0]** | **<0.001*** | **25.0 [23.0; 26.0]** | **20.5 [16.0; 24.0]** | **0.017*** |
| GDS | **3.5 [3.0; 4.3]** | **5.0 [4.0; 5.0]** | **0.023*** | 4.0 [3.0; 4.8] | 5.0 [3.5; 5.0] | 0.101 | **3.0 [2.0; 4.0]** | **5.0 [4.0; 5.0]** | **<0.001*** | 4.0 [2.3; 4.8] | 5.0 [3.0; 5.0] | 0.128 |
| CDR | **0.5 [0.5; 1.0]** | **1.0 [1.0; 1.0]** | **0.017*** | **0.5 [0.5; 1.0)** | **1.0 [1.0; 1.0]** | **0.032*** | **0.5 [0.5; 0.5]** | **1.0 [1.0; 1.0]** | **0.001*** | 0.5 [0.5; 1.0] | 1.0 [0.5; 1.0] | 0.121 |
| CDR Sum of Boxes | **3.3 [1.6-4.9]** | **5.3 [4.5; 6.5]** | **0.027*** | 3.5 [1.5; 5.5] | 5.0 [4.5; 8.0] | 0.069 | **2.0 [1.0; 3.3]** | **5.5 [4.5; 7.0]** | **<0.001*** | 3.75 [1.0; 5.1] | 5.0 [2.3; 7.5] | 0.087 |
| Evans index | 0.36 ± 0.04 | 0.38 ± 0.05 | 0.290 | 0.37 ± 0.04 | 0.37 ± 0.05 | 0.795 | 0.36 ± 0.04 | 0.37 ± 0.05 | 0.527 | 0.36 ±0.04 | 0.38 ± 0.05 | 0.349 |
| Callosal angle, degree | 96.3 ±16.3 | 87.5 ± 16.7 | 0.143 | 97.3 ± 16.4 | 87.4 ± 16.1 | 0.091 | 94.4 ± 18.5 | 91.6 ± 15.9 | 0.648 | 95.6 ± 17.0 | 90.4 ± 16.7 | 0.384 |
| 3^rd^ ventricle width, mm | 14.0 ± 3.1 | 13.9 ± 2.8 | 0.954 | 14.1 ± 3.2 | 13.7 ± 2.7 | 0.746 | 13.8 ± 3.4 | 14.0± 2.7 | 0.855 | 13.8 ± 3.1 | 14.0 ± 2.9 | 0.856 |

Data represent the mean ± standard deviation or median and interquartile ranges. Differences between groups were assessed using the χ2 test for dichotomous variables and the Student's t-test or Mann-Whitney U test for continuous variables..

Abbreviations: HTN, hypertension; DM, diabetes; MMSE, mini-mental status examination; GDS, global deterioration scale; CDR, clinical dementia rating; iNPHGS, idiopathic normal pressure hydrocephalus grading scale; GD, gait disturbance; CI, cognitive impairment; UD, urinary disturbance.

**Table S6. Diffusion tensor imaging (DTI) analysis in gait disturbance (GD) subgroups**

|  | | **FA** | | | **MD** | | | **AD** | | | **RD** | | |
| --- | --- | --- | --- | --- | --- | --- | --- | --- | --- | --- | --- | --- | --- |
| **ROIs** | | **low GD (N=18)** | **high GD**  **(N=15)** | **p** | **low GD**  **(N=18)** | **high GD**  **(N=15)** | **p** | **low GD**  **(N=18)** | **high GD (N=15)** | **p** | **low GD**  **(N=18)** | **high GD**  **(N=15)** | **p** |
| centrum semiovale | Lt | 0.372 ± 0.062 | 0.363 ± 0.066 | 0.696 | 0.856 [0.784;1.073] | 0.896 [0.772;1.033] | 0.986 | 1.191 [1.104;1.435] | 1.293 [1.046;1.446] | 0.986 | 0.686 [0.618;0.864] | 0.700 [0.598;0.824] | 1.000 |
|  | Rt | 0.369 ± 0.079 | 0.335 ± 0.061 | 0.179 | 0.913 ± 0.032 | 1.019 ± 0.215 | 0.093 | 1.282 ± 0.164 | 1.374 ± 0.225 | 0.182 | 0.728 ± 0.137 | 0.941 ±0.213 | 0.077 |
| corona radiata, anterior side | Lt | 0.322 ± 0.081 | 0.310 ± 0.086 | 0.690 | 1.001 [0.859;1.143] | 1.061 [0.920;1.151] | 0.464 | 1.404 ± 0.213 | 1.424 ± 0.174 | 0.773 | 0.804 [0.678;0.984] | 0.889 [0.743;0.991] | 0.509 |
|  | Rt | 0.306 ± 0.083 | 0.290 ± 0.079 | 0.556 | 0.943 [0.896;1.137] | 1.089 [0.955;1.217] | 0.401 | 1.358 ± 0.189 | 1.425 ± 0.229 | 0.362 | 0.800 [0.705;1.009] | 0.884 [0.778;1.062] | 0.381 |
| **corona radiata, lateral side** | **Lt** | **0.322 ± 0.059** | **0.273 ± 0.055** | **0.020*** | 1.161 ± 0.199 | 1.262 ± 0.176 | 0.136 | 1.564 ± 0.223 | 1.635 ± 0.208 | 0.357 | 0.959 ± 0.194 | 0.076 ± 0.169 | 0.078 |
|  | Rt | 0.270 ± 0.068 | 0.263 ± 0.060 | 0.765 | 1.197 ± 0.272 | 1.303 ± 0.202 | 0.219 | 1.536 ± 0.314 | 1.673 ± 0.227 | 0.168 | 1.028 ± 0.262 | 1.119 ± 0.196 | 0.275 |
| **Posterior limb of internal capsule** | **Lt** | 0.752 [0.714;0.768] | 0.740 [0.712;0.771] | 0.957 | 0.784 [0.763;0.837] | 0.746 [0.729;0.828] | 0.117 | **1.660 ± 0.090** | **1.579 ±0.119** | **0.034*** | 0.350 [0.320;0.394] | 0.334 [0.321;0.382] | 0.486 |
|  | **Rt** | 0.758 [0.724;0.776] | 0.749 [0.725;0.770] | 0.762 | **0.782 [0.776;0.801]** | **0.762 [0.732;0.777]** | **0.020*** | **1.626 [1.614;1.691]** | **1.574 [1.537;1.631]** | **0.044*** | 0.348 [0.324;0.385] | 0.337 [0.325;0.365] | 0.509 |
| anterior horn of lateral ventricle | Lt | 0.352 ± 0.065 | 0.316 ± 0.093 | 0.198 | 0.964 [0.899;1.067] | 0.915 [0.857;1.081] | 0.957 | 1.343 [1.194;1.456] | 1.274 [1.199;1.451] | 0.789 | 0.806 [0.670;0.902] | 0.769 [0.685;0.887] | 1.000 |
|  | Rt | 0.321 [0.296;0.400] | 0.286 [0.263;0.385] | 0.135 | 0.975 [0.941;1.128] | 0.991 [0.954;1.122] | 0.605 | 1.394 [1.285;1.464] | 1.385 [1.233;1.529] | 0.873 | 0.789 [0.717;0.966] | 0.839 [0.755;0.967] | 0.486 |
| **posterior horn of lateral ventricle** | Lt | 0.441 ± 0.055 | 0.396 ± 0.104 | 0.154 | 0.942 [0.822;1.124] | 1.094 [0.945;1.253] | 0.079 | 1.381 [1.280;1.585] | 1.612 [1.426;1.800] | 0.062 | 0.704 [0.599;0.872] | 0.856 [0.728;1.001] | 0.079 |
|  | **Rt** | 0.465 ± 0.078 | 0.440 ± 0.076 | 0.363 | **0.940 ± 0.188** | **1.078 ± 0.184** | **0.043*** | 1.440 ± 0.252 | 1.591 ± 0.191 | 0.066 | **0.690 ± 0.174** | **0.821 ± 0.193** | **0.049*** |
| corpus callosum | Genu | 0.720 [0.691;0.758] | 0.671 [0.553;0.782] | 0.532 | 0.923 [0.878;1.045] | 0.965 [0.867;1.218] | 0.605 | 1.887 [1.829;2.038] | 1.920 [1.810;2.022] | 0.901 | 0.472 [0.395;0.557] | 0.529 [0.374;0.798] | 0.682 |
|  | Splenium | 0.747 ± 0.113 | 0.729 ± 0.128 | 0.666 | 0.795 [0.765;0.970] | 0.831 [0.781;0.918] | 0.605 | 1.803 ± 0.196 | 1.816 ± 0.218 | 0.848 | 0.359 [0.272;0.559] | 0.394 [0.267;0.507] | 0.929 |

Data represent the mean ± standard deviation or median and interquartile ranges. Student t-test or Mann-Whitney U test were performed.

Abbreviations: DTI, diffusion tensor imaging; GD, gait disturbance; iNPHGS, idiopathic normal pressure hydrocephalus grading scale; ROIs, regions-of-interest; FA, fractional anisotropy; MD, mean diffusivity; AD, axial diffusivity; RD, radial diffusivity, Lt, left; Rt, right.

**Table S7. Diffusion tensor imaging (DTI) analysis in cognitive impairment (CI) subgroups**

|  | | **FA** | | | **MD** | | | **AD** | | | **RD** | | |
| --- | --- | --- | --- | --- | --- | --- | --- | --- | --- | --- | --- | --- | --- |
| **ROIs** | | **low CI**  **(N=14)** | **high CI**  **(N=19)** | **p** | **low CI**  **(N=14)** | **high CI**  **(N=19)** | **p** | **low CI**  **(N=14)** | **high CI**  **(N=19)** | **p** | **low CI**  **(N=14)** | **high CI**  **(N=19)** | **p** |
| **centrum semiovale** | Lt | 0.360 ± 0.071 | 0.373 ± 0.058 | 0.552 | 0.815 [0.749;0.994] | 0.946 [0.802;1.075] | 0.287 | 1.147 [1.043;1.394] | 1.316 [1.085;1.474] | 0.163 | 0.660 [0.610;0.784] | 0.719 [0.631;0.872] | 0.577 |
|  | **Rt** | **0.397 ± 0.069** | **0.322 ± 0.059** | **0.002*** | **0.888 ± 0.121** | **1.015 ± 0.199** | **0.043*** | 1.280 ± 0.152 | 1.357 ± 0.222 | 0.273 | **0.692 ± 0.124** | **0.844 ± 0.193** | **0.015*** |
| corona radiata, anterior side | Lt | 0.326 ± 0.081 | 0.310 ± 0.084 | 0.576 | 0.928 [0.854;1.148] | 1.038 [0.920;1.134] | 0.212 | 1.360 ± 0.173 | 1.453 ± 0.203 | 0.178 | 0.754 [0.656;0.984] | 0.866 [0.743;0.985] | 0.255 |
|  | Rt | 0.315 ±0.073 | 0.287 ± 0.086 | 0.338 | 0.926 [0.896;1.088] | 1.089 [0.956;1.217] | 0.106 | 1.325 ± 0.177 | 1.436 ± 0.220 | 0.133 | 0.753 [0.690;0.908] | 0.884 [0.791;1.062] | 0.163 |
| corona radiata, lateral side | Lt | 0.308 ± 0.076 | 0.293 ± 0.050 | 0.517 | 1.140 ± 0.206 | 1.256 ± 0.172 | 0.087 | 1.571 [1.380;1.685] | 1.727 [1.497;1.802] | 0.091 | 0.950 ± 0.210 | 1.058 ± 0.164 | 0.107 |
|  | Rt | 0.267 ±0.074 | 0.266 ± 0.056 | 0.971 | 1.188 ± 0.261 | 1.288 ± 0.231 | 0.251 | 1.522 ± 0.276 | 1.654 ±0.280 | 0.188 | 1.020 ± 0.262 | 1.105 ± 0.214 | 0.316 |
| **Posterior limb of internal capsule** | Lt | 0.764 [0.702;0.771] | 0.740 [0.714;0.767] | 0.602 | 0.798 ± 0.057 | 0.782 ± 0.073 | 0.522 | 1.656 ± 0.102 | 1.599 ± 0.112 | 0.141 | 0.346 [0.315;0.394] | 0.346 [0.322;0.395] | 0.872 |
|  | **Rt** | 0.765 [0.724;0.782] | 0.749 [0.725;0.762] | 0.321 | 0.783 [0.764;0.801] | 0.766 [0.741;0.779] | 0.091 | **1.642 [1.617;1.656]** | **1.613 [1.551;1.622]** | **0.019*** | 0.337 [0.316;0.385] | 0.338 [0.325;0.374] | 0.928 |
| **anterior horn of lateral ventricle** | **Lt** | **0.373 ± 0.072** | **0.308 ± 0.075** | **0.017*** | 0.928 [0.862;1.001] | 0.987 [0.862;1.086] | 0.483 | 1.313 [1.617;1.656] | 1.347 [1.174;1.475] | 0.679 | 0.763 [0.627;0.832] | 0.818 [0.719;0.896] | 0.199 |
|  | **Rt** | **0.361 ± 0.076** | **0.299 ±0.074** | **0.024*** | 0.966 [0.941;1.069] | 0.992 [0.995;1.151] | 0.271 | 1.395 [1.285;1.464] | 1.338 [1.268;1.526] | 0.986 | 0.767 [0.666;0.901] | 0.839 [0.786;0.986] | 0.142 |
| posterior horn of lateral ventricle | Lt | 0.452 ±0.076 | 0.397 ± 0.081 | 0.058 | 0.952 [0.798;1.128] | 1.055 [0.913;1.164] | 0.199 | 1.448 [1.262;1.669] | 1.520 [1.346;1.673] | 0.439 | 0.679 [0.565;0.926] | 0.777 [0.722;0.888] | 0.174 |
|  | Rt | 0.463 ± 0.074 | 0.446 ± 0.080 | 0.528 | 0.934 ± 0.196 | 1.053 ± 0.185 | 0.085 | 1.421 ± 0.258 | 1.573 ± 0.200 | 0.067 | 0.619 [0.566;0.833] | 0.799 [0.696;0.886] | 0.123 |
| corpus callosum | Genu | 0.730 [0.706;0.781] | 0.671 [0.580;0.758] | 0.132 | 0.942 [0.869;1.028] | 0.984 [0.874;1.268] | 0.321 | 1.905 [1.829;2.045] | 1.872 [1.815;2.028] | 0.928 | 0.461 [0.388;0.494] | 0.557 [0.392;0.828] | 0.212 |
|  | Splenium | 0.750 ± 0.125 | 0.730 ± 0.117 | 0.643 | 0.800 [0.730;0.970] | 0.831 [0.780;0.920] | 0.461 | 1.826 ± 0.214 | 1.797 ± 0.200 | 0.693 | 0.297 [0.251;0.559] | 0.394 [0.277;0.484] | 0.483 |

Data represent the mean ± standard deviation or median and interquartile ranges. Student t-test or Mann-Whitney U test were performed.

Abbreviations: DTI, diffusion tensor imaging; CI, cognitive impairment; iNPHGS, idiopathic normal pressure hydrocephalus grading scale; ROIs, regions-of-interest; FA, fractional anisotropy; MD, mean diffusivity; AD, axial diffusivity; RD, radial diffusivity, Lt, left; Rt, right.

**Table S8. Diffusion tensor imaging (DTI) analysis in urinary disturbance (UD) subgroups**

|  | | **FA** | | | **MD** | | | **AD** | | | **RD** | | |
| --- | --- | --- | --- | --- | --- | --- | --- | --- | --- | --- | --- | --- | --- |
| **ROIs** | | **low UD**  **(N=15)** | **high UD**  **(N=18)** | **p** | **low UD**  **(N=15)** | **high UD**  **(N=18)** | **p** | **low UD**  **(N=15)** | **high UD**  **(N=18)** | **p** | **low UD**  **(N=15)** | **high UD**  **(N=18)** | **p** |
| **centrum semiovale** | Lt | 0.367 ± 0.070 | 0.368 ± 0.058 | 0.966 | 0.880 [0.797;1.031] | 0.864 [0.752;1.074] | 0.986 | 1.220 [1.114;1.412] | 1.248 [1.050;1.469] | 0.986 | 0.701 [0.621;0.824] | 0.691 [0.603;0.869] | 0.735 |
|  | **Rt** | 0.363 ± 0.088 | 0.346 ± 0.058 | 0.501 | 0.937 ± 0.188 | 0.981 ± 0.175 | 0.484 | 1.305 ± 0.209 | 1.340 ± 0.190 | 0.614 | 0.752 ± 0.193 | 0.802 ± 0.174 | 0.444 |
| corona radiata, anterior side | Lt | 0.301 ± 0.085 | 0.330 ± 0.079 | 0.308 | 1.031 [0.863;1.279] | 1.026 [0.904;1.100] | 0.986 | 1.408 ± 0.226 | 1.418 ± 0.168 | 0.880 | 0.824 [0.678;1.120] | 0.804 [0.725;0.947] | 0.873 |
|  | Rt | 0.289 ± 0.086 | 0.307 ± 0.077) | 0.549 | 0.955 [0.873;1.242] | 1.001 [0.927;1.142] | 0.580 | 1.371 ± 0.254 | 1.404 ± 0.167 | 0.660 | 0.799 [0.698;1.079] | 0.835 [0.731;1.009] | 0.789 |
| corona radiata, lateral side | Lt | 0.307 ± 0.074 | 0.294 ± 0.050 | 0.553 | 1.152 ± 0.226 | 1.252 ± 0.153 | 0.142 | 1.531 ± 0.243 | 1.651 ± 0.179 | 0.115 | 0.963 ± 0.226 | 1.053 ± 0.148 | 0.179 |
|  | Rt | 0.255 ± 0.064 | 0.276 ± 0.062 | 0.339 | 1.240 ± 0.308 | 1.250 ± 0.188 | 0.912 | 1.568 ± 0.354 | 1.623 ± 0.213 | 0.600 | 1.076 ± 0.292 | 1.063 ± 0.184 | 0.877 |
| **Posterior limb of internal capsule** | Lt | 0.757 [0.700;0.766] | 0.746 [0.717;0.772] | 0.381 | **0.817 ± 0.068** | **0.766 ± 0.057** | **0.025*** | 1.649 ± 0.112 | 1.602 ± 0.107 | 0.228 | 0.353 [0.333;0.425] | 0.334 [0.320;0.382] | 0.155 |
|  | **Rt** | 0.759 [0.733;0.770] | 0.749 [0.724;0.776] | 0.789 | **0.779 [0.765;0.814]** | **0.764 [0.732;0.779]** | **0.016*** | **1.626 [1.615;1.673]** | **1.583 [1.547;1.636]** | **0.040*** | 0.340 [0.325;0.384] | 0.337 [0.324;0.381] | 0.532 |
| **anterior horn of lateral ventricle** | **Lt** | 0.359 ± 0.094 | 0.315 ±0.061 | 0.115 | 0.915 [0.846;1.034] | 0.979 [0.873;1.105] | 0.401 | 1.330 [1.212;1.403] | 1.327 [1.159;1.494]) | 0.735 | 0.761 [0.618;0.867] | 0.814 [0.740;0.890] | 0.215 |
|  | **Rt** | **0.366 ± 0.075** | **0.292 ±0.069** | **0.006*** | 0.966 [0.898;1.080] | 0.992 [0.962;1.245] | 0.166 | 1.389 [1.270;1.472] | 1.386 [1.282;1.567] | 0.762 | 0.777 [0.664;0.871] | 0.856 [0.777;1.084] | 0.067 |
| posterior horn of lateral ventricle | Lt | 0.413 ± 0.101 | 0.426 ± 0.066 | 0.664 | 1.094 [0.903;1.166] | 0.974 [0.850;1.104] | 0.229 | 0.585 [1.379;1.719] | 1.427 [1.270;1.612] | 0.166 | 0.860 [0.681;0.937] | 0.730 [0.633;0.859] | 0.274 |
|  | Rt | 0.460 ±0.083 | 0.447 ± 0.073 | 0.635 | 0.940 ± 0.155 | 1.055 ± 0.215 | 0.096 | 1.436 ± 0.206 | 1.569 ± 0.247 | 0.108 | 0.692 ± 0.154 | 0.797 ± 0.210 | 0.117 |
| corpus callosum | Genu | 0.716 [0.640;0.780] | 0.712 [0.630;0.758] | 0.817 | 0.950 [0.862;1.160] | 0.952 [0.882;1.045] | 0.929 | 1.938 [1.841;2.042] | 1.865 [1.809;1.999] | 0.464 | 0.443 [0.376;0.735] | 0.488 [0.395;0.627] | 0.735 |
|  | Splenium | 0.729 ± 0.156 | 0.747 ± 0.080 | 0.699 | 0.781 [0.762;0.997] | 0.839 [0.779;0.919] | 0.929 | 1.869 ± 0.233 | 1.759 ± 0.165 | 0.122 | 0.344 [0.250;0.588] | 0.384 [0.277;0.456] | 0.873 |

Data represent the mean ± standard deviation or median and interquartile ranges. Student t-test or Mann-Whitney U test were performed.

Abbreviations: DTI, diffusion tensor imaging; UD, urinary disturbance; iNPHGS, idiopathic normal pressure hydrocephalus grading scale; ROIs, regions-of-interest; FA, fractional anisotropy; MD, mean diffusivity; AD, axial diffusivity; RD, radial diffusivity, Lt, left; Rt, right.
